# Assessment of anxiety and quality of life in fibromyalgia patients

**DOI:** 10.1590/S1516-31802004000600005

**Published:** 2004-11-04

**Authors:** Tathiana Pagano, Luciana Akemi Matsutani, Elisabeth Alves Gonçalves Ferreira, Amélia Pasqual Marques, Carlos Alberto de Bragança Pereira

**Keywords:** Fibromyalgia, Quality of life, Anxiety, Questionnaires, Pain, Fibromialgia, Qualidade de vida, Ansiedade, Questionários, Dor

## Abstract

**CONTEXT:**

Fibromyalgia is a syndrome characterized by chronic, diffuse musculoskeletal pain, and by a low pain threshold at specific anatomical points. The syndrome is associated with other symptoms such as fatigue, sleep disturbance, morning stiffness and anxiety. Because of its chronic nature, it often has a negative impact on patients’ quality of life.

**OBJECTIVE:**

To assess the quality of life and anxiety level of patients with fibromyalgia.

**TYPE Of STUDY:**

Cross-sectional.

**SETTING:**

Rheumatology outpatient service of Hospital das Clínicas (Medical School, Universidade de São Paulo).

**METHODS:**

This study evaluated 80 individuals, divided between test and control groups. The test group included 40 women with a confirmed diagnosis of fibromyalgia. The control group was composed of 40 healthy women. Three questionnaires were used: two to assess quality of life (FIQ and SF-36) and one to assess anxiety (STAI). They were applied to the individuals in both groups in a single face-to-face interview. The statistical analysis used Student's t test and Pearson's correlation test (r), with a significance level of 95%. Also, the Pearson chi-squared statistics test for homogeneity, with Yates correction, was used for comparing schooling between test and control groups.

**RESULTS:**

There was a statistically significant difference between the groups (p = 0.000), thus indicating that fibromyalgia patients have a worse quality of life and higher levels of anxiety. The correlations between the three questionnaires were high (r = 0.9).

**DISCUSSION:**

This study has confirmed the efficacy of FIQ for evaluating the impact of fibromyalgia on the quality of life. SF-36 is less specific than FIQ, although statistically significant values were obtained when analyzed separately, STAI showed lower efficacy for discriminating the test group from the control group. The test group showed worse quality of life than did the control group, which was demonstrated by both FIQ and SF-36. Even though STAI was a less efficient instrument, it presented significant results, showing that fibromyalgia patients presented higher levels of anxiety, both on the state and trait scales. Thus, patients with fibromyalgia had higher levels of tension, nervousness, preoccupation and apprehension, and higher propensity towards anxiety.

**CONCLUSION:**

The three instruments utilized showed efficiency in evaluating fibromyalgia patients. FIQ was found to be the most efficient instrument for discriminating and assessing the impact of fibromyalgia on their quality of life. It can be concluded that such patients have a worse quality of life and higher levels of anxiety.

## RESUMO

**CONTEXTO:**

Fibromialgia é uma síndrome reumática caracterizada por dores músculo-esqueléticas difusas e crônicas e sítios dolorosos específicos à palpação, chamados de *tender points*, freqüentemente associados a fadiga, distúrbios do sono, rigidez matinal e, em alguns casos, dispnéia e ansiedade. Devido ao seu caráter crônico, a síndrome geralmente causa impacto negativo na qualidade de vida dos fibromiálgicos.

**OBJETIVO:**

Comparar a eficácia de instrumentos que avaliam a qualidade de vida de fibromiálgicos mensurada pelo Fibromyalgia Impact Questionnaire (FIQ) e pelo Medical Outcomes Study 36-item Short-Form Healthy Survey (SF-36), e a ansiedade avaliada pelo Inventário de Ansiedade Traço-estado (IDATE).

**TIPO DE ESTUDO:**

Transversal.

**LOCAL:**

Ambulatório de Reumatologia do Hospital das Clínicas da Faculdade de Medicina da Universidade de São Paulo (HC/FMUSP).

**MÉTODOS:**

Participaram do estudo 80 sujeitos: 40 com fibromialgia (grupo teste) e 40 saudáveis (grupo controle). Três questionários (dois para avaliação de qualidade de vida – FIQ e SF-36 –; e um para ansiedade – IDATE) foram aplicados aos indivíduos dos dois grupos em uma única entrevista. Toda a análise estatística foi realizada utilizando-se o Teste "t" Student e o teste de Correlação de Pearson (r), com significância p < 0,05. Além disso, o teste estatístico Qui-quadrado de Pearson, para homogeneidade, foi usado para comparar o grau de escolaridade entre os grupos teste e controle.

**RESULTADOS:**

Os resultados obtidos mostram que houve diferença estatisticamente significante entre os grupos (p = 0,00), indicando que os fibromiálgicos têm pior qualidade de vida e níveis mais altos de ansiedade. A correlação entre os três questionários foi alta (r = 0,90).

**DISCUSSÃO:**

O impacto negativo na qualidade de vida decorrente da Fibromialgia tem sido relatado em muitos estudos, nos quais os protocolos de avaliação são os principais istrumentos de medida. O FIQ é um instrumento utilizado em vários estudos clínicos para avaliar a função física. Este estudo comprovou a eficiência do FIQ para avaliar o impacto da fibromialgia sobre a qualidade de vida. O SF-36 é menos específico que o FIQ, mas também se mostrou eficiente para a avaliação da qualidade de vida de fibromiálgicos, uma vez que os discrimina dos indivíduos saudáveis. A ansiedade é conside-rada um sintoma secundário da fibromialgia e é freqüentemente grave nos casos de fibromialgia. Um dos instrumentos utilizados para avaliá-la é o IDATE. No presente estudo, apesar de apresentar resultados estatisticamente significantes quando analisado isoladamente, o IDATE mostrou-se menos eficaz que os demais instrumentos para distinguir o grupo teste do controle. Tratando-se mais especificamente da qualidade de vida, foi possível confirmar que o grupo teste apresenta pior qualidade de vida quando comparado ao controle. Isso foi demonstrado pelos dois instrumentos utilizados para avaliar a qualidade de vida (FIQ e SF-36). Quanto à ansiedade, apesar do IDATE ter sido o instrumento menos eficaz, pôde-se observar resultados significantes na avaliação dos grupos teste e controle, evidenciando que os pacientes com fibromialgia apresentam maior nível de ansiedade, tanto no estado como no traço. Portanto, os pacientes com fibromialgia têm níveis mais elevados de tensão, nervosismo, preocupação e apreensão (avaliados pela escala A-estado); e maior propensão à ansiedade (avaliada pela escala A-traço).

**CONCLUSÃO:**

Os três instrumentos utilizados mostraram ser eficazes para avaliar pacientes fibromiálgicos, porém o FIQ mostrou ser o mais eficaz para discriminar e avaliar o impacto da fibromialgia na qualidade de vida. Conclui-se que os pacientes fibromiálgicos têm pior qualidade de vida e níveis mais altos de ansiedade.

## INTRODUCTION

Fibromyalgia is a rheumatic syndrome that can be characterized by chronic and diffuse musculoskeletal pain, and also by a low pain threshold at specific anatomical points named tender points. The syndrome is also often associated with other symptoms such as fatigue, sleep disturbance, morning stiffness^1^ and occasionally with dyspnea and anxiety.^2-5^ The syndrome mostly affects women between 40 and 55 years old.

The main symptom in fibromyalgia is diffuse and chronic pain. Sometimes the pain can be so severe that it interferes in the individual's work, day-to-day activities and quality of life.^6-8^ The chronic pain causes a component of suffering, with many contributory factors such as anxiety, frustration and anger; it also has an adverse impact on the person's mood.^9^ Such symptoms lead to disability and end up having a negative impact on the patient's life.^10^ Therefore, it is important to measure this impact.

Anxiety is an important factor to be taken into consideration when talking about fibromyalgia.^11,12^ In one study developed by Yunus et al., 70% of the patients considered themselves anxious, and in 68% of the patients the symptoms were worsened due to anxiety and mental stress.^13^

The use of quality of life assessment instruments and questionnaires has been recognized as an important source of scientific knowledge in the healthcare field. In clinical practice, they can identify patients’ needs and assess intervention effectiveness.

Quality of life assessment instruments can be generic or specific. In 1991, Burckhardt et al.^14^ proposed and tested an instrument for assessing quality of life in fibromyalgia, the Fibromyalgia Impact Questionnaire (FIQ). They concluded that FIQ is valid to be used in research or clinical situations.

White et al.,^10^ using FIQ, reported that fibromyalgia caused a negative impact on the quality of life of patients of an economically productive age. This resulted from symptoms such as fatigue and subjective weakness, in addition to pain. These symptoms caused disability that contributed towards these individuals’ incapacity to work and consequently resulted in reduced family income and worsened quality of life.

A generic instrument for quality of life assessment that is widely used is the *Medical Outcomes Study 36-item Short-Form Health Survey* (SF-36).^15^ It was translated and validated for use in Portuguese by Ciconelli^16^ and has been used as a generic measure for quality of life assessment in other situations, such as knee arthroplasty,^17^ rheumatoid arthritis^16^ and osteoarthritis.^18^

Anxiety, mentioned by some authors^11,12^ as one of the symptoms in fibromyalgia, can be measured by the State-Trait Anxiety Inventory (STAI). STAI was proposed by Spielberger and Gorsuch^19^ in 1964, with the intention of generating a direct research instrument for self-evaluation that could be used to measure traits and states of anxiety among normal adults.^20^

## PURPOSE

The objective of this study was to assess the quality of life and anxiety level of fibromyalgia patients, using FIQ, SF-36 and STAI.

## METHODS

**Subjects** – The study included 80 individuals. Forty of them had a diagnosis of fibromyalgia according to the American College of Rheumatology criteria,^1^ recruited from the Rheumatology outpatient service of Hospital das Clínicas, Medical School of Universidade de São Paulo, and the other 40 were healthy individuals forming a control group. The subjects were selected according to the following criteria: age between 35 and 60 years; adequate cognitive level for understanding the procedures and following the guidance given; agreement to participate in the study; and signing of the informed consent document.

**Setting -** The study was performed by the Physical Therapy Department, within the Rheumatology outpatient service of Hospital das Clínicas, Medical School, Universidade de São Paulo.

**Procedures** – All the individuals in both groups were evaluated at a single face-to-face interview. The following data was obtained: personal data (age, sex, race, weight and height); previous and present occupation; level of education; marital status; history of pain: when it had occurred, painful sites on the body (in decreasing order of intensity), period of the day when it was most severe and factors that would increase or decrease the pain; and their quality of sleep and mattress and pillow type. Because of patients’ difficulties in reading, the investigators decided to read the questionnaires along with them.

Quality of life assessment was done by means of the following instruments: *Fibromyalgia Impact Questionnaire* (FIQ),^14^ *Medical Outcomes Study 36-item Short-Form Health Survey* (SF-36)^15^ and *State-Trait Anxiety Inventory* (STAI).^19^ FIQ involves questions related to functional capacity, professional situation, psychological disturbances and physical symptoms. The higher the score, the higher the impact of fibromyalgia is on quality of life. SF-36 is a generic multidimensional instrument that assesses eight characteristics: functional capacity, physical characteristics, pain, general health, vitality, social and emotional characteristics and mental health. In addition, anxiety was assessed using STAI, which evaluates traits and states of anxiety. STAI consists of two distinct anxiety scales: trait scale (A-trait) and state scale (A-state). Both scales are composed of 20 questions and require that the subjects describe how they feel generically, on the A-trait scale, and how they feel at a specific moment, on the A-state scale.

**Statistical analysis** – The statistical analyses used Student's t statistics for comparing groups and for testing Pearson correlations. Also, the Pearson Chi-squared statistics test for homogeneity, with Yates correction, was used for comparing scholarity between Test and Control groups. To avoid working directly with all the questionnaire items, we reduced their dimensions to real numerical values. We named these reductions the quality of life indices. The FIQ and SF-36 reductions were called FIQI and SFI, respectively. In addition, there are differences between the two questionnaires regarding the final score evaluation: for FIQ, the higher the final value, the worse the quality of life is; and for SF-36, the higher the final value, the better the quality of life is. To construct FIQI, seven items were taken into consideration (variables evaluated by the instrument), while eight items were considered for SFI. FIQI was calculated as 10 minus the mean of the seven items, while SFI was calculated as the mean of the eight items, divided by 10. By doing this, both FIQI and SFI vary from 0 to 10, and both indicate that the higher their values are, the better the quality of life is. The same was done for STAI, for which the index was called IDI, so that comparisons between the three instruments would be possible.

Finally, to illustrate the results described above, we constructed a dispersion diagram for the indexes, thereby emphasizing the two groups. On the same diagram, we plotted a diagonal line that contains the points where the indices are equal.

## RESULTS

Table 1 shows the patients’ demographic data. There were no statistically significant demographic differences between the groups. This demonstrated that the sample was homogeneous and therefore would allow valid comparisons between the indexes for the groups. Participants’ educational levels varied greatly, with statistically significant difference (p = 0.03). Only 12.5% of the test group and 40% of the control group had a college degree. All participants were females.

**Table 1:** Demographic data of 80 participants in a study on fibromyalgia in São Paulo (40 patients and 40 controls)

	Control group Mean/SD	Test group Mean/SD	P
Age (years)	49.48 ± 7.65	49.43 ± 5.95	0.97
Weight (kg)	65.25 ± 12.73	67.62 ± 13.62	0.42
Height (m)	1.60 ± 0.06	1.58 ± 0.07	0.27
BMI (kg/m^2^)	25.29 ± 4.39	26.93 ± 5.06	0.12
Schooling:			
No education	0%	5%	
Incomplete elementary school	2.5%	27.5%	
Completed elementary school	17.5%	17.5%	
Incomplete high school	10%	12.5%	
Completed high school	30%	25%	
College	40%	12.5%	
Female Sex	100 %	100 %	

*SD = standard deviation; BMI = body mass index. Note: in Brazil, elementary school comprises eight years and high school, the following three.*

### Questionnaire evaluation

The three questionnaires were applied to all subjects, thus totaling 240 questionnaires. The results obtained are shown in Tables 2, 3 and 4.

The results obtained via FIQ (Table 2) showed statistically significant differences between the control and the test groups, for all variables. The only exception was the variable "work missed", since both groups had zero values.

**Table 2:** Data from the Fibromyalgia Impact Questionnaire (FIQ) applied to 40 patients with fibromyalgia and 40 controls in São Paulo

Variables	Control group	Test group	"t" test	
	Mean ± SD	Mean ± SD	t	p
1) Physical function	4.35 ± 5.16	14.93 ± 5.37	8.98	0.00
2) Feeling well	6.33 ± 1.53	1.93 ± 1.85	11.62	0.00
3) Work missed	0 ± 0	0 ± 0	––––	––––
4) Job ability	0.2 ± 0.65	7.36 ± 2.02	21.33	0.00
5) Pain	0.66 ± 1.46	7.77 ± 1.72	19.9	0.00
6) Fatigue	2.37 ± 2.99	7.7 ± 2.21	9.03	0.00
7) Morning tiredness	1.39 ± 2.5	7.18 ± 2.8	9.75	0.00
8) Stiffness	0.16 ± 0.82	6.97 ± 2.47	16.54	0.00
9) Anxiety	3.54 ± 3.4	7.71 ± 2.68	6.09	0.00
10) Depression	1.7 ± 2.34	6.23 ± 3.26	7.14	0.00

*For α = 0.05, "t" (t á/2) = 2.10.*

Table 3 shows the results obtained via SF-36. There were statistically significant differences between the control and test groups for all variables. Only the variable "role - emotional" had a lower value than the others, although it still showed a statistically significant difference.

**Table 3:** Data from the Medical Outcomes Study 36-item Short Form Health Survey (SF-36) applied to 40 patients with fibromyalgia and 40 controls in São Paulo

Variables	Control group	Test group	"t" test	
	Mean ± SD	Mean ± SD	t	p
1) Physical function	86.63 ± 16.5	33.00 ± 19.57	13.25	0.00
2) Role - physical	90.00 ± 22.5	9.38 ± 22.42	16.05	0.00
3) Body pain	76.65 ± 20.78	30.23 ± 14.83	11.5	0.00
4) General health	81.47 ± 17.78	45.65 ± 21.65	8.09	0.00
5) Vitality	67.93 ± 20.37	33.13 ± 21.26	7.47	0.00
6) Social function	82.94 ± 22.38	44.00 ± 26.48	7.11	0.00
7) Role - emotional	77.53 ± 37.27	30.83 ± 38.79	5.49	0.00
8) Mental health	75.7 ± 14.84	45.6 ± 22.13	7.14	0.00

*For α = 0.05, "t" (t _α/2_) = 2.10.*

The results obtained via STAI (Table 4) showed a statistically significant difference between the control and test groups. Variables such as "anxiety state" and "anxiety trait" had higher mean values in the test group than in the control group. There was no intra-group difference between STAI trait and STAI state.

**Table 4:** Data from the State-Trait Anxiety Inventory (STAI)) applied to 40 patients with fibromyalgia and 40 controls in São Paulo

Anxiety variable	Control group	Test group	"t" test	
	Mean ± SD	Mean ± SD	t	p
Anxiety-state	35.13 ± 12.11	51.88 ± 11.68	6.30	0.00
Anxiety-trait	36.78 ± 9.44	53.18 ± 12.6	6.60	0.00

*For α = 0.05, "t" (t _α/2_) = 2.10.*

### Questionnaire efficacy evaluation

To better illustrate the results shown in Tables 2, 3 and 4, dispersion diagrams were constructed between FIQI and SFI, between FIQI and IDI and, finally, between SFI and IDI. In these diagrams, a diagonal line was traced out where the points are equal: FIQI = SFI, FIQI = IDI and SFI = IDI.

**Figure 1:**
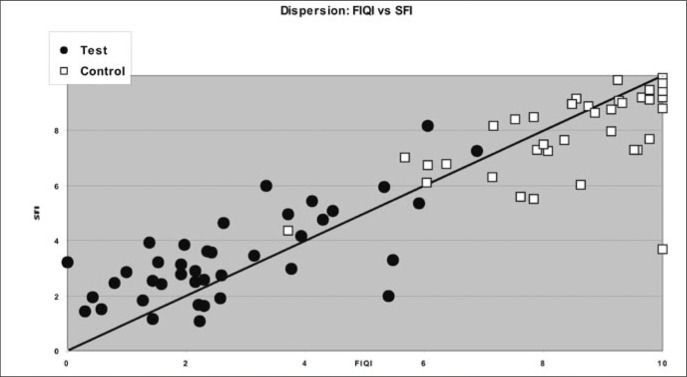
Dispersion of results between the Fibromyalgia Impact Questionnaire Index (FIQI) and the Medical Outcomes Study 36-item Short Form Health Survey (SF-36) Index (SFI) in a sample of 80 individuals in São Paulo.

Figure 1 shows the dispersion for FIQI versus SFI. It can be seen that the control group is more homogeneous than the test group, since its values are closer to the line. The dispersion in the control group is smaller, since its variation limits are seen to be more restricted than for the test group. The points for the control group are concentrated in the upper right part of the graph, thereby indicating that they are high-value points for FIQI as well as for SFI. On the other hand, the points for the test groups are concentrated in the lower left part of the graph, thereby indicating lower values for FIQI as well as for SFI. In comparing the dispersion results within the same group, it is noticeable that the dispersion is smaller in FIQI than in SFI.

**Figure 2:**
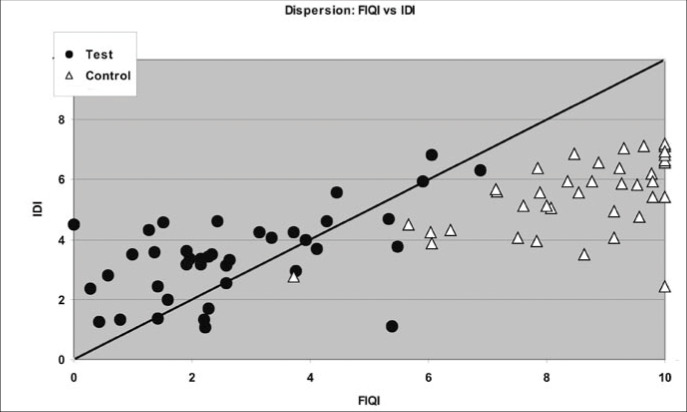
Dispersion of results between the Fibromyalgia Impact Questionnaire Index (FIQI) and the State-Trait Anxiety Inventory Index (IDI) in a sample of 80 individuals in São Paulo.

Figure 2 shows the dispersion graph for FIQI versus IDI. Two different populations can be distinguished: one concentrated mostly in the upper right quadrant, representing the control group; and another one, concentrated in the lower left quadrant, representing the test group. The test group presents lower values via both instruments (FIQ and STAI). The opposite is seen for the control group. In comparing the dispersion results within the same group, it is noticeable that the dispersion is smaller in FIQI than in IDI.

**Figure 3:**
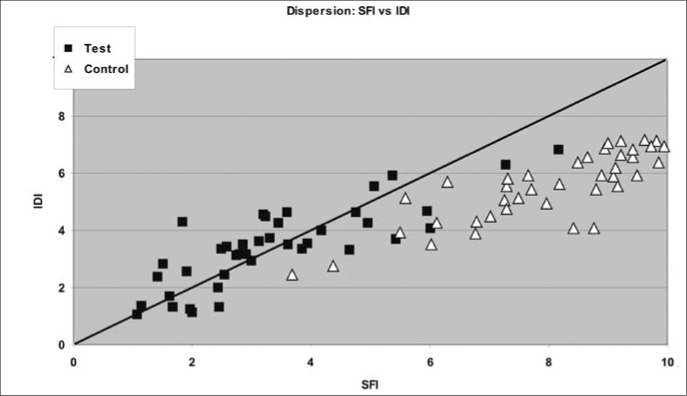
Dispersion of results between the Medical Outcomes Study 36-item Short Form Health Survey (SF-36) Index (SFI) and the State-Trait Anxiety Inventory Index (IDI) in a sample of 80 individuals in São Paulo.

Figure 3 shows the dispersion graph for SFI versus IDI. It can be seen that there is a slight concentration of the control group in the lower right quadrant, while the test group is concentrated in the upper left quadrant. The test group has lower values for SFI as well for IDI, and greater dispersion on the graph than does the control group. In comparing the dispersion results within the same group, it is noticeable that the dispersion is smaller in SFI than in IDI. The points that are below the line have lower power for discriminating fibromyalgia.

**Figure 4:**
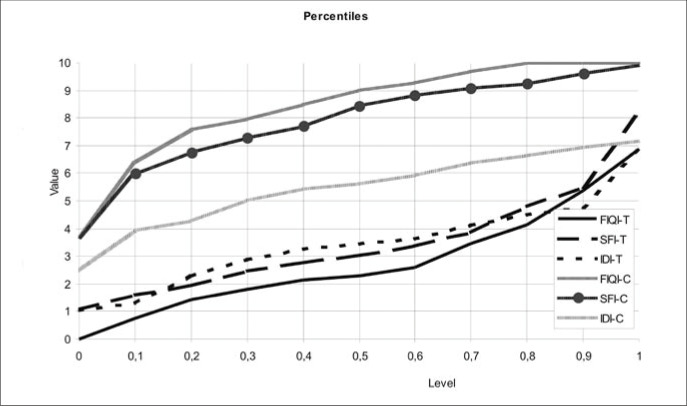
Percentiles of the results of quality of life evaluation by three questionnaires applied to 40 fibromyalgia patients and 40 healthy controls. FIQ = Fibromyalgia Impact Questionnaire; SF-36 = Medical Outcomes Study 36-item Short Form Health Survey; STAI = State-Trait Anxiety Inventory. FIQI-T = FIQ Index — Test; SFI-T = SF-36 Index — Test; IDI-T = STAI Index — Test; FIQI-C = FIQ Index — Control; SFI-C = SF-36 Index — Control; IDI-C = STAI Index — Control.

In Figure 4, a percentile graph illustrates the conclusions of the study. Dotted lines represent the control group, while continuous lines represent the test group. The instruments showed efficiency in distinguishing the test group from the control group. For example, at percentile 50, it can be seen that FIQ is a better instrument than SF-36, which in turn is better than STAI for evaluating individuals with fibromyalgia. This is justified by the bigger difference between the test and the control curves when FIQ is used, and also by the smaller difference when using STAI. However, Figure 4 also shows that the two groups are very different. For instance, at percentile 80 the test group has value 4, while in the control group these values are between 9 and 10 in FIQ and SF-36, and 7 in STAI.

## DISCUSSION

The negative impact on the quality of life caused by fibromyalgia has been reported from many trials in which assessment protocols were the major evaluation instruments.^21-24^

FIQ is an instrument that has been used in various clinical trials for assessing physical function. It also helps in the measurement of the efficacy of the therapeutic intervention in fibromyalgia cases, and it has been specifically validated for assessing the quality of life among fibromyalgia patients/individuals.^14^

Trials that used FIQ as the assessment protocol have attested to its efficacy in making comparisons with healthy subjects^21^ and with subjects affected by other diseases,^24,25^ as well as between fibromyalgia patients before and after treatment programs, including physical therapy,^26-30^ and in prospective trials.^31^ Similarly, this study has confirmed the efficacy of FIQ for evaluating the impact of fibromyalgia on the quality of life.

SF-36 is another questionnaire that assesses quality of life, but it is less specific than FIQ. It is capable of assessing the relationship between health status and quality of life: its eight subscales have a high correlation with functional disability. The majority of these subscales represent aspects of health that are important for fibromyalgia patients or the diffuseness of the pain. The gravity of functional impairment, as assessed by SF-36, differentiates such patients from normal individuals, as well as differentiating patients with fibromyalgia from those with diffuse pain only. The SF-36 questionnaire is also efficient at assessing quality of life among fibromyalgia patients, since it can distinguish healthy individuals from those with fibromyalgia, as previously described by Martinez et al.^23^

By assessing health status and physical function, these questionnaires also allow psychological evaluation of such patients, for example, through their anxiety and depression subscales.^32^ Anxiety is considered to be a common secondary symptom of fibromyalgia, and it is frequently severe in fibromyalgia cases.^25^ One of the instruments used for evaluating anxiety is STAI,^19,25,33^ which was proposed for measuring the trait (propensity towards anxiety) and the state (tension, nervousness, preoccupation and apprehension) of anxiety. STAI thus allows measurement of the level of psychological disturbance among subjects that is mainly related to anxiety. In the present study, although statistically significant values were obtained when they were analyzed separately, STAI showed lower efficacy for discriminating the test group from the control group, in comparison with other instruments. Such a finding is a general reflection of the fact that both populations presented considerable anxiety levels, which makes it more difficult to characterize the subpopulations on the basis of these variables.

More specifically regarding quality of life, it was possible to confirm that the test group showed worse quality of life than did the control group, which was demonstrated by both instruments used for assessing quality of life (FIQ and SF-36). This result is comparable with what other studies have suggested, in which patients with chronic diseases such as fibromyalgia may have lower quality of life levels than seen in healthy populations.^34^

With regard to anxiety, even though STAI was a less efficient instrument, it presented significant results in assessing both the test and control groups, showing that fibromyalgia patients presented higher levels of anxiety, both on the state and trait scales. Thus, patients with fibromyalgia had higher levels of tension, nervousness, preoccupation and apprehension (assessed via the A-state scale), and higher propensity towards anxiety (evaluated via the A-trait scale).

From the results presented, although the three instruments showed values that differed by statistically significant amounts, it was possible to notice a difference in efficacy between them. Thus, it can be concluded that FIQ, which involves specific questions for fibromyalgia related to functional capacity, professional condition, psychological disturbances and physical symptoms, showed more efficiency in assessing the impact of fibromyalgia on the quality of life of patients with fibromyalgia, since it characterized the test group better and allowed better discrimination between the test and the control groups. These findings can be seen illustrated in the dispersion and percentile graphs. This instrument has already been used in other trials, which attests to its efficacy.^14, 24,26-28^

SF-36 was the second most effective instrument in differentiating the test group from the control group, also on the basis of these graphs. SF-36 is an instrument for assessing the generic quality of life, and it evaluates functional capacity, physical characteristics, pain, general health status, vitality, social and emotional characteristics and mental health. The results show that SF-36 also demonstrated efficiency in assessing the quality of life of fibromyalgia patients, with results similar to those found by Ciconelli^16^ and Dias^18^. In another study, Brazilian women with fibromyalgia from the Sorocaba region, State of São Paulo, were compared with a control group in relation to quality of life effects. In this, SF-36 was utilized as an assessment tool and it was also concluded that the disease has a negative impact on quality of life.^35^

The strong correlation between the three questionnaires shows that all of them are capable of distinguishing subjects with fibromyalgia from healthy ones. FIQ was the most efficient questionnaire for characterizing fibromyalgia, followed by SF-36 and then STAI (Figure 4). This suggests that FIQ may be the instrument of preference when only assessing populations with fibromyalgia. This result was expected, since FIQ was specially constructed for assessing patients with fibromyalgia. Related trials, in which SF-36 was compared to specific questionnaires, have also reported greater efficacy of the specific questionnaire. Nevertheless, the discriminatory capacity of SF-36 must be stressed.^36,37^

The results found in this trial have confirmed the efficacy of the instruments in assessing the quality of life and anxiety among subjects with fibromyalgia. They have also reinforced the credibility of the questionnaires as tools for assessing multiple aspects of fibromyalgia and for following up these characteristics over a period of time and/or after therapeutic intervention. These instruments may be important in comparative studies among patients with fibromyalgia and other diseases, in relation to healthy subjects. Comparative studies of this nature are essential for establishing the impact of fibromyalgia on healthcare systems and in drawing up extensive and interdisciplinary treatment programs for fibromyalgia patients. Specifically in the field of physical therapy, questionnaires allow the assessment of the results obtained by those planned treatments.

## CONCLUSION

The group of patients with fibromyalgia presented worse quality of life when compared with the control group. This showed that fibromyalgia interferes in quality of life and anxiety levels, in relation to trait as well as to state of anxiety, which suggests that these symptoms may be generated by fibromyalgia.

By comparing the three questionnaires – FIQ, SF-36 and STAI – it was possible to conclude that all of them are efficient, not only in assessing quality of life, but also for assessing anxiety. Nevertheless, because FIQ is a specific instrument, it was the best at distinguishing the individuals with fibromyalgia, through showing values that had greater statistical significance.
